# Expression Plasticity of Peroxisomal Acyl-Coenzyme A Oxidase Genes Implies Their Involvement in Redox Regulation in Scallops Exposed to PST-Producing *Alexandrium*

**DOI:** 10.3390/md20080472

**Published:** 2022-07-24

**Authors:** Moli Li, Yangrui Wang, Zhihong Tang, Huizhen Wang, Jingjie Hu, Zhenmin Bao, Xiaoli Hu

**Affiliations:** 1 MOE Key Laboratory of Marine Genetics and Breeding, College of Marine Life Sciences, Ocean University of China, Qingdao 266003, China; lml940520@163.com (M.L.); wangyangrui@stu.ouc.edu.cn (Y.W.); tangzhihong@ouc.edu.cn (Z.T.); hujingjie@ouc.edu.cn (J.H.); zmbao@ouc.edu.cn (Z.B.); hxl707@ouc.edu.cn (X.H.); 2 Laboratory for Marine Fisheries Science and Food Production Processes, Qingdao National Laboratory for Marine Science and Technology, Qingdao 266237, China; 3 Laboratory of Tropical Marine Germplasm Resources and Breeding Engineering, Sanya Oceanographic Institution, Ocean University of China, Sanya 572000, China

**Keywords:** scallops, ACOX, gene expansion, paralytic shellfish toxins, *Alexandrium*

## Abstract

Filter-feeding bivalves can accumulate paralytic shellfish toxins (PST) produced by toxic microalgae, which may induce oxidative stress and lipid peroxidation. Peroxisomal acyl-coenzyme A oxidases (ACOXs) are key enzymes functioning in maintaining redox and lipid homeostasis, but their roles in PST response in bivalves are less understood. Herein, a total of six and six *ACOX*s were identified in the *Chlamys farreri* and *Patinopecten yessoensis* genome, respectively, and the expansion of *ACOX1*s was observed. Gene expression analysis revealed an organ/tissue-specific expression pattern in both scallops, with all *ACOX*s being predominantly expressed in the two most toxic organs, digestive glands and kidneys. The regulation patterns of scallop *ACOX*s after exposure to different PST-producing algaes *Alexandrium catenella* (ACDH) and *A. minutum* (AM-1) were revealed. After ACDH exposure, more differentially expressed genes (DEGs) were identified in *C*. *farreri* digestive glands (three) and kidneys (five) than that in *P*. *yessoensis* (two), but the up-regulated DEGs showed similar expression patterns in both species. In *C*. *farreri*, three DEGs were found in both digestive glands and kidneys after AM-1 exposure, with two same *CfACOX1*s being acutely and chronically induced, respectively. Notably, these two *CfACOX1*s also showed different expression patterns in kidneys between ACDH (acute response) and AM-1 (chronic response) exposure. Moreover, inductive expression of *CfACOX*s after AM-1 exposure was observed in gills and mantles, and all DEGs in both tissues were up-regulated and their common DEGs exhibited both acute and chronic induction. These results indicate the involvement of scallop *ACOX*s in PST response, and their plasticity expression patterns between scallop species, among tissues, and between the exposure of different PST analogs.

## 1. Introduction

Organisms are constantly affected by various stresses from the environment, which can trigger a series of cellular and systemic events that affect their survival and adaptation [1]. Oxidative stress induced by the generation of excess reactive oxygen species (ROS) has emerged as a critical factor for organisms to respond to environmental stresses. The most typical responses to oxidative stress involve distinct organelles, including mitochondria, lysosomes, and peroxisome [2,3,4], among which peroxisome are primarily known for their role in cellular lipid metabolism, and are also increasingly recognized as potential regulators of oxidative stress-related signaling pathways as many peroxisomal enzymes catalyze redox reactions as part of their normal function [4].

Peroxisomal acyl-coenzyme A oxidases (ACOXs) are the rate-limiting enzymes that catalyze the initial step of the β-oxidation system in the peroxisome and are always used as biomarkers for peroxisome proliferation [5]. They can catalyze the α,β-dehydrogenation of acyl-CoA by transferring electrons in the form of H^−^ from their prosthetic group FADH_2_ to O_2_, thereby generating H_2_O_2_, which is detoxified by catalase [6]. ACOXs are generally classified into three subtypes: ACOX1, ACOX2, and ACOX3. The difference among these three enzyme types is that ACOX1 can only desaturate straight-chain acyl-CoA; ACOX2 mainly acts on branched-chain acyl-CoA; ACOX3 recognizes different acyl-CoAs with or without a 2-methyl branch [7]. Studies have shown that *ACOX*s are not only essential for fatty acid oxidation and redox homeostasis but also play an important role in the response to oxidative stress caused by environmental xenobiotics. For example, in Chinese toad, cadmium exposure triggered oxidative stress and down-regulated the expression of *ACOX* in the liver [8]. The up-regulation of ACOX protein was observed in sea cucumber after a diet with tussah immunoreactive substances (TIS) [9]. Furthermore, bisphenol A (BPA) exposure caused an imbalance in the redox state in the liver of common carp and up-regulated the expression of lipid metabolism-related genes, including *ACOX1*, in the liver [10].

For aquatic organisms, increased toxic stresses in habitats have a direct impact on the suitability and survival of their populations. Harmful algal blooms (HABs) are an important source of toxic stress in aquatic habitats [11]. During HABs, filter-feeding bivalves can accumulate paralytic shellfish toxins (PST) produced by marine microalgae, especially the dinoflagellates of the genus *Alexandrium* [12]. PST are acute neurotoxins and include at least 57 derivatives of saxitoxin (STX) [13], which can reversibly bind voltage-gated Na^+^ channels (NaV) of excitable cell membranes, thus blocking the conduction of nerve signals and causing neuromuscular disorders [14,15]. Although bivalves could tolerate high concentrations of PST due to possessing toxin-resistant amino acids in NaV protein [14,16], PST accumulation also induces oxidative stress, resulting in an imbalance between the production of ROS and the antioxidant system [17,18,19]. Studies on the mechanisms of oxidative stress caused by PST in bivalves are extensive but mainly focus on the antioxidant-related genes, such as the genes encoding superoxide dismutase (SOD), glutathione peroxidase (GPx), and glutathione S-transferase (GST), which were found to be regulated in response to PST exposure [20,21,22]. However, whether the peroxisomal β-oxidation pathway, which is essential for redox homeostasis, is involved in PST response remains poorly studied.

Among bivalves, scallops have an excellent ability to accumulate a higher level of PST, and can retain these toxins for a longer time than other members [23], making them to be an ideal species to study the toxin stress and tolerance of bivalves. To investigate the effect of PST exposure on the peroxisomal β-oxidation system, we systematically identified the *ACOX* genes in *Chlamys farreri and Patinopecten yessoensis*, the main commercial species of scallops cultured in China [16,24]. We also analyzed the expression responses of *ACOX* genes after exposure to two different PST-producing *Alexandrium* (*A. catenella* and *A. minutum*). Our results revealed the expansion of scallop *ACOX* genes, and their toxin-, tissue- and species-specific expression pattern during toxic algae challenges. Our findings will expand the understanding of *ACOX* functions in scallops and can guide future studies focusing on the role of peroxisomal β-oxidation and its interactions with PST.

## 2. Results and Discussion

### 2.1. Identification of ACOXs in Scallop Genome

A total of six and six *ACOX* genes were identified in *C. farreri* *(CfACOX)* and *P. yessoensis* (*PyACOX*) genomes, respectively (Table S1). Basic information regarding their genome position, CDS length, protein length, ACOX domain region, isoelectric point (pI), and molecular weight (MW) were summarized in Table 1. The coding sequences of *CfACOXs* ranged from 1896 to 2187 bp and encoded proteins from 631 to 728 amino acids (aa), whereas *PyACOXs* varied from 1989 to 2187 bp encoding 662 to 728 aa. The predicted MW of ACOX proteins ranged from 70.84 kDa to 81.47 kDa with the pIs ranging from 5.74 to 8.89 in *C. farreri*, and 74.51 kDa to 81.36 kDa with pIs from 5.81 to 8.86 in *P. yessoensis*.

In *C. farreri*, we identified four *ACOX1*s, one *ACOX2*, and one *ACOX3*, and also obtained four *ACOX1*s, one *ACOX2*, and one *ACOX3* in *P. yessoensis* (Table 2). Notably, the numbers of *ACOX1*s in scallops and oyster (three copies) genomes were more than that in vertebrates (one copy), and owl limpet (one copy), implying the expansion of *ACOX1*s in bivalves. We also found the gene expansion event in *Caenorhabditis elegans*, but the expanded genes in this species were annotated as *ACOX1/2*. In addition, *ACOX2* was not found in zebrafish, while *ACOX3* was absent from fruit fly, which was possibly a result of species-specific losses.

### 2.2. Conserved Structures of ACOX Genes in C. farreri and P. yessoensis

**Figure 1:**
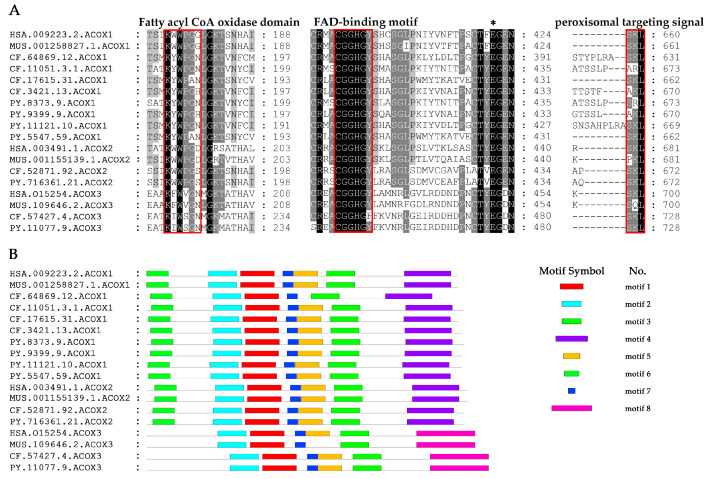
Analysis of conserved protein structures presented in scallop ACOX proteins compared with their vertebrate homologs. (**A**) Alignments of conserved structural domains of CfACOXs and PyACOXs. The fatty acyl CoA oxidase (ACOX) domain, FAD-binding motif, and peroxisomal targeting signal (PTS) are labeled with red frames, while the conserved glutamate is indicated with asterisk (*). (**B**) The conserved motifs of CfACOXs and PyACOXs. Each colored box represents a motif in the protein. (HSA: *Homo sapiens*, MUS: *Mus musculus*, CF: *Chlamys farreri*, PY: *Patinopecten yessoensis*).

Sequence alignment of scallop ACOX proteins with their homologs in other selected species revealed the presence of conserved structural domains (Figure 1A and Figure S1), including the fatty acyl CoA oxidase (ACOX) domain (K-W/F-W-I/V/P-G-G/N/D), FAD-binding motif (CGGHGY), and peroxisomal targeting signal (PTS) [7]. Near the N-terminal of scallop ACOXs, a conserved stretch of six amino acids (K-Y/W/I-W-P/S-G/A-N/H/S) is present, proposed as an ACOX motif. The ACOX domain of ACOX1 is more similar to ACOX2 than ACOX3, but all of them contain conserved Trp (W), which contribute to the stabilization of the isoalloxazine ring of FAD through hydrogen bonds [25]. Like all other ACOXs, FAD-binding motif was highly conserved in scallops, as well as among different ACOX members. Behind the FAD-binding motif, a conserved glutamate, which constitutes the catalytic site involved in the α-proton abstraction from the substrate [26], is also found in scallop ACOXs. In addition, the tripeptide SKLs or a related sequence (S/A/V/P-K/R/H/Q/N-L/I/M) at its carboxy-terminal end was shown to be the targeting signal for many peroxisomal matrix proteins [27]. All scallop ACOX proteins contained the PTS, with ACOX2 and ACOX3 containing SKL, and ACOX1 members containing SKL or several variants (S/A-K/R-L).

We also found the motifs of scallop ACOX proteins were highly conserved (Figure 1B). Almost all CfACOX1 and PyACOX1 members contained seven conserved motifs, which showed high similarity in terms of the type, order, and the number of motifs with their vertebrate homologs. Similar results were also observed in ACOX2 and ACOX3. In addition, the motifs of scallop ACOX1 and ACOX2 members were more similar than that of ACOX3. ACOX1 and ACOX2 shared all seven motifs, while only five motifs were shared with ACOX3. These results indicate that scallop ACOX members probably have analogous functions to other vertebrate counterparts.

### 2.3. Phylogenetic Relationship of ACOXs between Bivalves and Other Organisms

**Figure 2:**
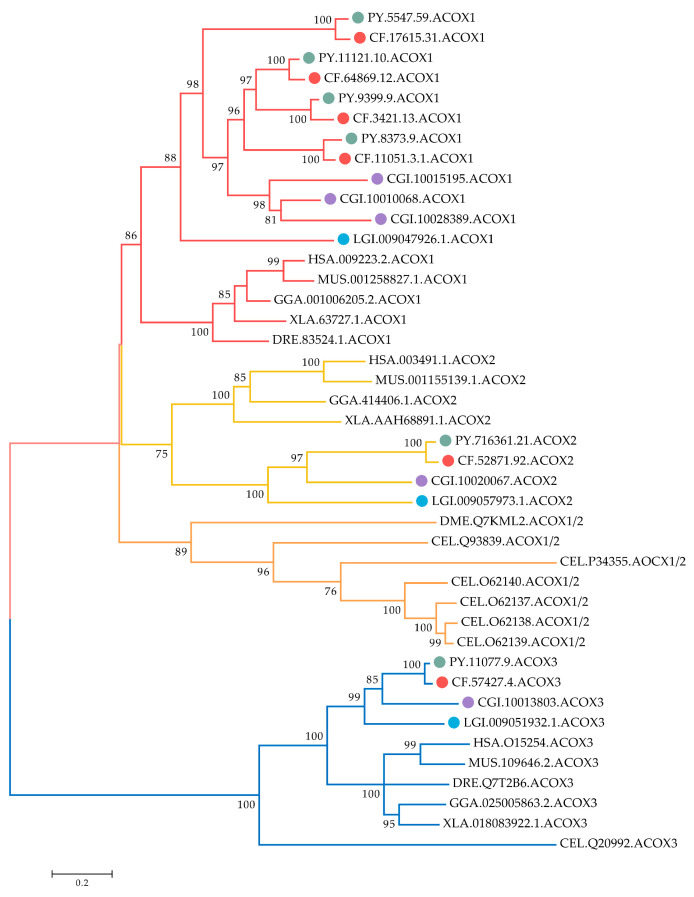
Phylogenetic tree of ACOX proteins from *C. farreri*, *P. yessoensis*, and other selected organisms. The tree was constructed using the maximum-likelihood (ML) method with LG + G + I module. Numbers at the branch point of the node represent the value resulting from 1000 replications. CfACOX, PyACOX, *Crassostrea gigas* ACOX, and *Lottia gigantean* ACOX proteins are marked with red, green, purple, and blue dots, respectively. Branches of ACOX1, ACOX2, and ACOX3 proteins are highlighted in red, yellow, and blue, respectively. HAS: *H. sapiens*, MUS: *M. musculus*, GGA: *Gallus gallus*, XLA: *Xenopus laevis*, DRE: *Danio rerio*, DME: *D. melanogaster*, CEL: *C. elegans*, LGI: *L. gigantean*, CGI: *C. gigas*, CF: *C. farreri*, PY: *P. yessoensis*. The accession numbers of ACOXs used in the phylogenetic analysis are listed in Table S2.

Phylogenetic analysis of ACOX amino acid sequences from 11 selected species was conducted. As shown in Figure 2, the scallop ACOX members can be classified into two major branches. One branch contained vertebrate ACOX1 and ACOX2 members, as well as their corresponding ACOX orthologous in mollusks, fruit fly, and nematode. Each member of ACOX1 and ACOX2 in scallops clustered into well-supported separate clades with its orthologues of other species. Several *C. elegans* ACOX1/2 and a *Drosophila melanogaster* ACOX1/2 formed another sub-clade and were rooted at the clade of ACOX1 and ACOX2, indicating they shared the same ancestor. This finding also implies that *ACOX1* and *ACOX2* were produced before the speciation of mollusks. Notably, species-specific expansion of *ACOX1*s was observed in bivalves. In the bivalve specific group, several pairs of *C. farreri* and *P. yessoensis* ACOX1s were closely associated and clustered with *C. gigas*. Given that lineage-specific gene expansion is often associated with the emergence of new biological functions and a response to diverse environmental pressures [28], the high number of bivalve *ACOX1*s derived from bivalve-specific expansion suggests that these genes may play a major role in bivalve’s adaptation to the stressful marine environment. The other branch was dominated by ACOX3 members, and scallop ACOX3s were well distributed with their corresponding ACOX3 orthologous from other species, suggesting that they are evolutionarily conserved.

### 2.4. Expression Profiles of Scallops ACOXs in Developmental Stages and Adult Organs/Tissues

**Figure 3:**
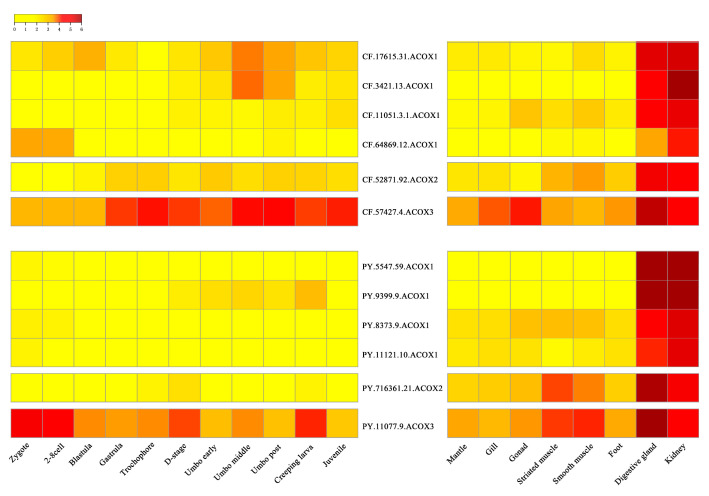
Heatmap of *ACOX* gene expression profiles of *C. farreri* and *P. yessoensis* during developmental stages and in adult organs/tissues. The expression levels, as represented by log_2_ (RPKM + 1) values, are shown in the gradient heat map with colors ranging from yellow (low expression) to red (high expression).

According to the RNA-Seq data, we found all *CfACOX* and *PyACOX* genes were expressed (RPKM > 1) in at least one of the selected developmental stages or organs/tissues (Figure 3, Table S3). During the development stages of scallops, most *ACOX*s, including several *ACOX1*s (*CF.17615.31* and *CF.64869.12* in *C. farreri*, *PY.5547.59*, *PY.8373.9* and *PY.11121.10* in *P. yessoensis*), one *ACOX2* and one *ACOX3* in both scallops, showed widespread expression from zygote to larvae and juvenile stages, indicating their maternal origin played an important role in maintaining the fatty acid metabolic balance during development. The other two *CfACOX1*s (*CF.3421.13* and *CF.11051.3.1*) and one *PyACOX1* (*PY.9399.9*) started to be abundantly expressed from the blastula or D stage veliger, and sustained their expression in the following umbo larvae and juvenile stages, implying the involvement of these *ACOX1*s during scallop metamorphosis and post-larval development.

In adult organs/tissues, the organ/tissue-specific expression patterns of *ACOX*s were found in both scallops and all *CfACOX*s and *PyACOX*s were predominantly expressed in both digestive glands and kidneys (Figure 3, Table S3). For expanded *ACOX*1s including four *CfACOX1*s and four *PyACOX1*s, the highest expression level was observed in the kidneys followed by the digestive glands, while *ACOX2* and *ACOX3* genes were the most abundantly expressed in the digestive glands, followed by the kidneys, and showed relatively lower expression in other organs/tissues. Abundant *ACOX* transcripts in kidneys and digestive glands have also been found in other species [29]. The kidneys and digestive glands were reported to be the crucial metabolic and defense organs against oxidative stress, the dominant *ACOX*s expression in these two tissues suggested that scallop *ACOX*s might play crucial roles in the molluscan metabolism and defense system.

### 2.5. Expression Regulation of Scallops ACOXs after Toxic Dinoflagellates Exposure

**Figure 4:**
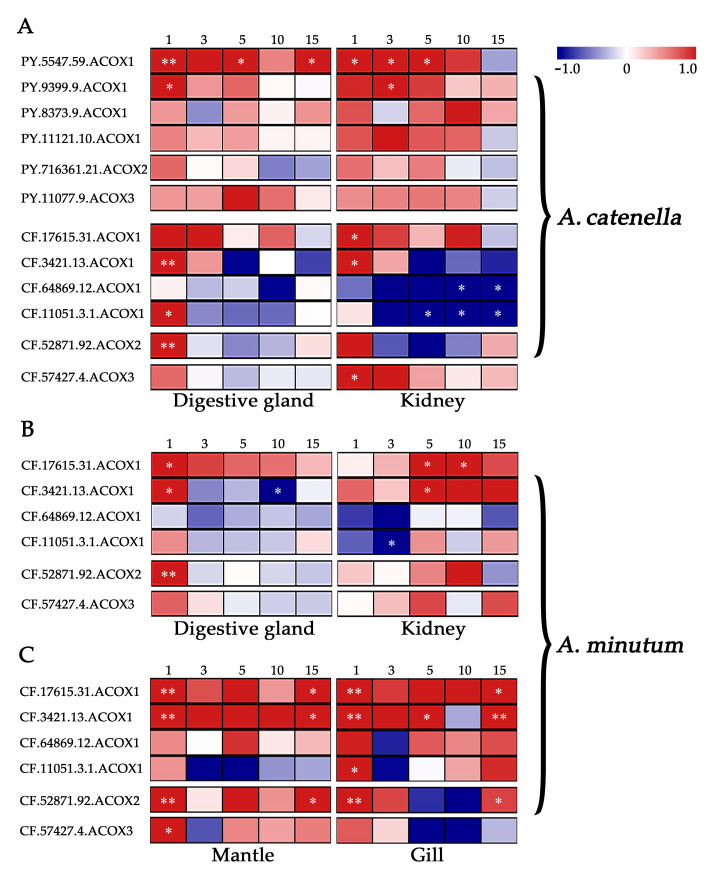
Temporal expressions of *PyACOX*s and *CfACOX*s in digestive glands and kidneys after exposure to (**A**) *Alexandrium catenella* (ACDH) and (**B**) *A. minutum* (AM-1), and (**C**) that in mantles and gills after exposure to *A. minutum* (AM-1). The heatmap was based on log_2_FC values. The exposure time (1, 3, 5, 10, or 15 days) is displayed above the heatmap. * represents significant regulation with |log_2_FC| > 1 and *p*-Value < 0.05, ** represents very significant regulation with |log_2_FC| > 1 and FDR < 0.05.

To evaluate the possible involvement of scallop *ACOX*s in response to PST-producing algae challenge, we examined their expression changes after exposure to toxic dinoflagellates *Alexandrium* (Figure 4, Table S4). Dinoflagellates of the genus *Alexandrium* are the major PST producers in scallop farming, but the toxicities and harmful mechanisms of different *Alexandrium* species are varied [30]. We first analyzed the expression profile of *ACOX*s in the digestive glands and kidneys of two scallop species (*P. yessoensis* and *C. farreri*) after exposure to *A. catenella* (strain ACDH), which mainly produces N-sulphocarbamoyl derivatives (C1/2) [30]. In scallops, the digestive glands and kidneys showed different roles in toxin metabolism, with the former primarily responsible for absorbing PST directly from algae, and the latter mainly responsible for transforming toxins into higher toxic analogs [16]. In *P. yessoensis*, the two same *PyACOX1*s (*PY.5547.59* and *PY.9399.9*) were significantly up-regulated (|log_2_Fold Change (FC)| > 1 and *p* < 0.05) in both digestive glands and kidneys (Figure 4A). These two differentially expressed genes (DEGs) exhibited similar expression patterns, with *PY.5547.59* showing both acute and chronic induction, and *PY.9399.9* showing acute response in both tissues. By contrast, more *ACOX*s were regulated in *C. farreri* after ACDH challenge. In digestive glands, two *CfACOX1*s (*CF.3421.13* and *CF.11051.3.1*) and one *CfACOX2* showed significant up-regulation, while all the *CfACOX*s except *CfACOX2* showed significant alteration in kidneys with two *CfACOX1*s (*CF.17615.31* and *CF.3421.13*) and *CfACOX3* being up-regulated and the other two *CfACOX1*s (*CF.11051.3.1* and *CF.64869.12*) being down-regulated (Figure 4A). The DEGs in both tissues showed similar expression patterns, with all up-regulated DEGs being acutely induced on day 1. Although different types of PST were contained in digestive glands (mainly C1/C2) and kidneys (mainly transformed type NeoSTX) after ACDH exposure [31], the similar expression patterns of *CfACOX*s indicate that the toxic effects produced by C1/C2 and NeoSTX might have similar effects on the β-oxidation pathway. For down-regulated DEGs in kidneys, the chronic response was observed on days 5, 10, and 15 post exposure.

As the strong response in *C. farreri* to ACDH, we further investigated the transcriptional responses of *ACOX*s in this species after exposure to the other toxic dinoflagellates, *A. minutum* (strain AM-1) (Figure 4B), which mainly produces PST analogs of gonyautoxins (GTXs, mainly GTX1-4) with higher toxicity [30]. After AM-1 exposure, two *CfACOX1*s (*CF.17615.31* and *CF.3421.13*) and one *CfACOX2* were identified to be differentially expressed in digestive glands, while three *CfACOX1*s (*CF.17615.31*, *CF.3421.13,* and *CF.11051.3.1*) were significantly regulated in kidneys (|log_2_FC| > 1 and *p* < 0.05). Two of these DEGs (*CF.17615.31* and *CF.3421.13*) were regulated in both tissues, but they exhibited different expression patterns, being acutely up-regulated on day 1 in digestive glands, while showing chronic induction on day 5 or day 10 in kidneys. Considering the difference in toxin profiles between digestive glands (mainly GTX1-4) and kidneys (containing transformed type STX) after AM-1 exposure [16,31], the organ/tissue-specific pattern of *CfACOX*s might be due to the different response mechanisms to GTXs and STX challenges. Compared to the results of ACDH exposure, we found *CfACOX*s expression patterns were diverse between AM-1 and ACDH challenges in the kidneys. All up-regulated DEGs were acutely induced after ACDH exposure, whereas they showed chronic induction after AM-1 exposure (Figure 4A, B). This time-specific expression pattern might be related to the toxicity levels of PST in kidneys. By contrast, *CfACOX*s exhibited similar acute responses in digestive glands with both diets.

Furthermore, we also observed the inductive expression of *CfACOX*s after AM-1 exposure in mantles and gills (Figure 4C), the two organs having relatively large contact areas with the surrounding water and playing crucial roles in water filtering and toxin absorption [32,33,34]. All DEGs in both tissues were up-regulated after AM-1 challenge, with two *CfACOX1*s (*CF.17615.31* and *CF.3421.13*), one *CfACOX2*, and one *CfACOX3* showing significant alteration in mantles, while three *CfACOX1*s (*CF.17615.31*, *CF.3421.13,* and *CF.11051.3.1*) and one *CfACOX2* being significantly up-regulated in gills (|log_2_FC| > 1 and *p* < 0.05). Notably, three DEGs (*CF.17615.31*, *CF.3421.13*, and *CF.52871.92*) were found in both tissues, and they exhibited similar expression patterns with exhibiting both acute (day 1) and chronic responses (day 15) to AM-1, which was different from the results in digestive glands and kidneys. It is worth noting that one *ACOX1* member (*CF.3421.13*) in *C. farreri* was up-regulated in all tissues after both *Alexandrium* exposure, which could be a useful indicator of the peroxisomal β-oxidation pathway under algal toxin stress in bivalves.

Overall, the expression changes in *ACOX*s were widely observed in four tissues of scallops after exposure to different toxic algae, suggesting the involvement of the peroxisomal β-oxidation pathway in PST response. Most *ACOX*s, especially the expanded *ACOX1*s, were up-regulated after toxic algae challenge, which also indicates the activation of the β-oxidation pathway. In addition, we found diversified responsive patterns of scallop *ACOX* genes between different scallop species, between the exposure of different dinoflagellates, as well as among tissues, which implies the expression plasticity of scallop *ACOX*s in response to the stress caused by PST-producing algae. Our findings will provide comprehensive information for understanding the adaptive evolution and functional diversity of *ACOX*s in scallops.

## 3. Materials and Methods

### 3.1. Identification and Sequence Analysis of ACOX Genes in C. farreri and P. yessoensis

Firstly, we used the available sequences of *ACOX*s from invertebrates (*D. melanogaster* and *C*. *elegans*) and vertebrates (*H. sapiens*, *M. musculus*, *G. gallus*, *X. laevis*, and *D. rerio*) as queries to search against the transcriptomes of two scallops (*P. yessoensis* and *C. farreri*) to obtain candidate *ACOX* sequences [16,24]. The candidate *ACOX*s sequences were translated through the ORF finder (https://www.ncbi.nlm.nih.gov/orffinder/, accessed on 15 March 2022). Then, the predicted ACOX proteins were aligned to the public databases including KEGG (https://www.kegg.jp/, accessed on 18 March 2022), UniProt (http://www.uniprot.org/, accessed on 18 March 2022), and the NCBI non-redundant (Nr) protein sequence database with BLASTP (e-value set: 1E-05). The sequences with significant blast hit to known ACOX proteins were obtained as candidates and further verified, whether or not they contained ACOX domain, by the Conserved Domains Database (https://www.ncbi.nlm.nih.gov/cdd, accessed on 25 March 2022) and SMART tools (http://smart.embl-heidelberg.de/, accessed on 25 March 2022). Similar methods were used to identify *ACOX* genes in *C. gigas* and *L. gigantean* [35,36]. The compute pI/MW tool (https://web.expasy.org/compute_pi/, accessed on 5 April 2022) was used to predict the pI value and MW (kDa). “Multiple EM for Motif Elicitation” (MEME) version 5.4.1 (https://meme-suite.org, accessed on 15 April 2022) was employed to compare conserved motifs among scallop ACOX proteins with other organisms.

### 3.2. Multiple Alignment and Phylogenetic Analysis

The ACOX protein sequences from four mollusks and other representative species were employed for phylogenetic analysis. Multiple alignments of ACOX proteins were performed using Clustal Muscle [37]. The maximum likelihood (ML) phylogenetic tree was constructed using MEGA 6.0 with the best fit model LG + G + I model (LG model and Gamma distribution with Invariant sites) and 1000 bootstrap pseudo-replicates [38].

### 3.3. Expression Analysis of ACOX Genes during Developmental Stages and in Adult Organs/Tissues

The spatiotemporal expression profiles of *C. farreri* and *P. yessoensis ACOX* genes in different developmental stages and adult organs/tissues (at least three biological replicates in each sample) were obtained from published RNA-seq data [16,24]. Developmental stage samples include zygotes, 2–8 cells, blastula, gastrula, trochophore, d-stage larvae, umbo early larva, umbo middle larva, umbo post larva, creeping larva, and juvenile scallop. Samples of adult organs/tissues include mantle, gill, gonad, striated muscle, smooth muscle, foot, digestive gland, and kidney. The expression of all *ACOX* genes was normalized and represented in the form of reads per kilobase of exon model per million mapped reads (RPKM). A custom R script was used to generate a heatmap with the log_2_(RPKM+1) value.

### 3.4. Expression Analysis of ACOX Genes Exposed to Toxic Dinoflagellates

In order to analyze the effect of PST-producing *Alexandrium* on the expression of *ACOX* genes in scallops, we challenged the *C. farreri* and *P. yessoensis* through exposure to the *A. catenella* (strain ACDH) and/or *A. minutum* (strain AM-1). These two toxic strains of *Alexandrium* were cultured independently in F/2 medium under a light–dark cycle of 14h:10h and were collected when the cell density reached 3 × 10^5^ cells/mL in the exponential growth phase [39,40]. Before the feeding experiment, the PST profiles of two toxic strains were measured by high-performance liquid chromatography with tandem mass spectrometry (HPLC-MS/MS) analysis following the methods described in our previous study [41]. The main types of PST in ACDH and AM-1 were N-sulphocarbamoyl derivatives (C1/C2) and gonyautoxins (GTXs, mainly GTX1-4), respectively, and no other noxious metabolites were previously reported in these two strains [41]. *C. farreri* and *P. yessoensis* were acclimated in filtered and aerated seawater at 12.5 ± 0.5 °C for three weeks and then maintained independently with aeration during the exposure experiments. Each scallop was fed 3 L of *Alexandrium* with a density of 2500 cells/mL once a day. Three individuals were randomly collected on days 0, 1, 3 (acute response), 5, 10, and 15 (chronic response) of exposure. The digestive glands, kidneys, gills, and mantles of these scallops were dissected, washed with sterile seawater, and then stored in a −80 °C ultra-low temperature environment.

Total RNA was extracted using the conventional guanidinium isothiocyanate method [42]. RNA-seq libraries were constructed using the NEB Next mRNA Library Prep Kit and subjected to PE125 sequencing on the Illumina HiSeq2000 platform. RNA-seq reads were mapped to *C. farreri* and *P. yessoensis* genomes using Tophat 2.0.9 [43]. The expression of all *ACOX* genes was normalized and represented in the form of RPKM. Fold change (FC) for each test time point was calculated as log_2_FC between toxin-exposed and control groups. Significantly DEGs were identified using the edgeR package [44] with statistically significant cutoff of |log_2_FC| > 1 and *p*-Value < 0.05, and the very significantly DEGs with cutoff of |log_2_FC| > 1 and corrected FDR value < 0.05. A heatmap was generated with the log_2_FC values using Multiple Experiment Viewer 4.9.0 software (https://sourceforge.net/projects/mev-tm4/files/mev-tm4/MeV%204.9.0/, accessed on 26 April 2022).

## 4. Conclusions

In the present study, we performed the first systematic analysis of *ACOX* genes in bivalve mollusks. Our data revealed the expansion of *ACOX*s in bivalve genomes, and their considerable structural diversity, conserved domains and motifs, as well as conserved evolutionary relationships. The organ/tissue-specific expression pattern of scallop *ACOX*s was also revealed, which was predominantly expressed in digestive glands and kidneys. Furthermore, we found that most of *ACOX*s, especially the expanded *ACOX1*s, showed significant alteration after toxic algae challenge, and the regulatory patterns of *ACOX*s presented scallop species-, dinoflagellate strain- and tissue-dependent expression patterns.

## Figures and Tables

**Table 1:** Basic information of scallop *ACOX* genes.

Gene	Chromosome No.	Scaffold ID	CDS (bp)	Amino Acid (aa)	ACOX Pfam Position	Isoelectric Point (pI)	Molecular Weight (kDa)
*CF.17615.31.ACOX1*	Chr10	17615	1989	662	477–658	8.42	74.92
*CF.3421.13.ACOX1*	Chr2	3421	2013	670	479–660	8.89	75.31
*CF.11051.3.1.ACOX1*	Chr10	11051	2022	673	481–662	8.75	75.11
*CF.64869.12.ACOX1*	Chr10	64869	1896	631	437–619	8.40	70.84
*CF.52871.92.ACOX2*	Chr7	52871	2019	672	484–665	7.82	75.26
*CF.57427.4.ACOX3*	Chr9	57427	2187	728	536–722	5.74	81.47
*PY.5547.59.ACOX1*	Chr18	5547	1989	662	477–658	8.86	74.70
*PY.9399.9.ACOX1*	Chr18	9399	2013	670	479–660	8.64	74.51
*PY.8373.9.ACOX1*	Chr18	8373	2022	673	481–662	8.65	75.36
*PY.11121.10.ACOX1*	Chr18	11121	2010	669	473–655	7.26	75.20
*PY.716361.21.ACOX2*	Chr6	716361	2019	672	484–665	7.55	75.71
*PY.11077.9.ACOX3*	Chr10	11077	2187	728	536–722	5.81	81.36

**Table 2:** Gene number comparison of *ACOX* genes among selected vertebrates, *Drosophila melanogaster*, *Caenorhabditis elegans*, and mollusk genomes.

Species	*ACOX1*	*ACOX2*	*ACOX3*	Total
*Homo sapiens*	1	1	1	3
*Mus musculus*	1	1	1	3
*Gallus gallus*	1	1	1	3
*Xenopus laevis*	1	1	1	3
*Danio rerio*	1	0	1	2
*Lottia gigantean*	1	1	1	3
*Crassostrea gigas*	3	1	1	5
*Patinopecten yessoensis*	4	1	1	6
*Chlamys farreri*	4	1	1	6
*Drosophila melanogaster*	1 (*ACOX1/2*)		0	1
*Caenorhabditis elegans*	6 (*ACOX1/2*)		1	7

## Data Availability

Not applicable.

## Supplementary Materials

The following supporting information can be downloaded at: https://www.mdpi.com/article/10.3390/md20080472/s1, Figure S1: Multiple sequences alignments of the deduced amino acid sequence of scallop ACOXs and their vertebrate homologs; Table S1: Sequences of ACOX proteins in *C. farreri*, *P. yessoensis*, *C. gigas*, and *L. gigantea*; Table S2: The accession numbers of *ACOX*s used in phylogenetic analysis.; Table S3: The average RPKM of *ACOX*s in developmental stages and adult organs/tissues; Table S4: The log_2_(FC), *p* and FDR values of scallop *ACOX*s after toxic *A. catenella* and *A. miutum* exposure at different time points.
