# Supplementary material for: Expression Plasticity of Peroxisomal Acyl-Coenzyme A Oxidase Genes Implies Their Involvement in Redox Regulation in Scallops Exposed to PST-Producing Alexandrium
Source: Mar Drugs. 2022 Jul 24;20(8):472. doi: 10.3390/md20080472 (PMC9332717; doi:10.3390/md20080472)
Supplement: Supplementary file 1 [file marinedrugs-20-00472-s001.zip › Figure S1.pdf]

```
HSA.009223.2.ACOX1     : -----------------MNP---------------------D-----LRRERDSASFNPELLTHILDGSPEKTRRRREIENMILNDPDF-QHEDLNFLTRSQRYEVAVRKSAIMVKKMREFGIA :  80
                                                                                                                                                 *
MUS.001258827.1.ACOX1  : -----------------MNP---------------------D-----LRKERAAATFNPELITHILDGSPENTRRRREIENLILNDPDF-QHEDYNFLTRSQRYEVAVKKSATMVKKMREFGIA :  80
CF.64869.12.ACOX1      : ---------MAAAKKMSVNP---------------------D-----LAKERTKATFDVEQLTYILYLGKERTKRKRYLEDLAVNDPVIKECKPWAFCSREEQYEMGLKKHIHIAKQIKLLGIT :  89
CF.11051.3.1.ACOX1     : -------MASAGTGTTAVNP---------------------D-----LAKERRNASFHVENLTNFIYHGPERVRRRRYMQNIALQEPALKDMKPWAFRTREEENELSLCKQLHVLKRQKELGIT :  91
CF.17615.31.ACOX1      : -------------MAVAVNP---------------------D-----LERERQNATFDRETLTDFLYGGAQKTQRRRYLKNVVFSDPEYQTMKSRVFCNREELYALHLQKYNIMRQIQEKLHIT :  85
CF.3421.13.ACOX1       : ---------MAARGRVTVNA---------------------D-----LAKERASATFDVENLTNFIDGGVAATKRKRYLQDLAINDPELKNSKPWAVMTREEQYETALRKHIYMTKRLEEMGIT :  89
PY.8373.9.ACOX1        : -------MASAGTGKMTVNP---------------------D-----IAKERRNAGFHVENLTNFIYHGPERVRRRRYMQNIALQDPVMKDSKPWAFRTREEEYELSLRKQLHVLQRQKDLGIT :  91
PY.9399.9.ACOX1        : ---------MAARGRVTVNA---------------------D-----LAKERTSATFDVENLTHFIDGGVAATKRKRYLQDLAINDPELKNSKPWAVMTRKEQYETALRKHIYMAKRLEEMGIT :  89
PY.11121.10.ACOX1      : ---------------MNVNP---------------------D-----LAKERSKATFDVEQLTYVLYLGKERTERKRYLENLAVNDPVIKECKPWAFCSREEQYEMALKKHIHIAKQIKLLGFT :  83
PY.5547.59.ACOX1       : -------------MASAVNP---------------------D-----LERERKRATFDRETLTNFLYGGAQKKQRRRYLKNAVFSDPEYQAMKSRVFCSREELYALHLQKYSIFRRIQENLNIT :  85
HSA.003491.1.ACOX2     : MGSPVHRVSLGDTWSRQMHP---------------------D-----IESERYMQSFDVERLTNILDGGAQNTALRRKVESIIHSYPEF-SCKDNYFMTQNERYKAAMRRAFHIRLIARRLGWL :  97
MUS.001155139.1.ACOX2  : MGNPGDRVSLGETWSREVHP---------------------D-----IDSERHSPSFSVERLTNILDGGIPNTELRRRVESLIQRDPVF-NLKHLYFMTRDELYEDAVQKRFHLEKLAWSLGWS :  97
CF.52871.92.ACOX2      : ----MANEVMAGMGSTAINP---------------------D-----IARERLKASFDPVELTHVLDGGKERTARRKELENLLFSDPLF-CLRDNIYLSTSDLYSATMARSVKLFRLKKDQKWS :  93
PY.716361.21.ACOX2     : ----MANEVMAGMESNAINP---------------------D-----IARERLKATFDPLELTHIFDGGKERTTRRKELENLLFSDPLF-CLRDNIYLSTSDLYSATMARSVKLFRLKQDQKWS :  93
HSA.O15254.ACOX3       : ------MASTVEGGDTALLP---------------------EFPRGPLDAYRARASFSWKELALFTE-GEGMLRFKKTIFSALENDPLF-ARSPGADLSLEKYRELNFLR----CKRIFEYDFL :  91
MUS.109646.2.ACOX3     : ------MGSLPEEKDSALWS---------------------DTPKGPLSAYRARASFNSGELLLFWD-GQDVIHFKKTIFSTLENDPLF-ARSYGADLPLEKLRELNFLR----CKRVFEYGFF :  91
CF.57427.4.ACOX3       : MGDQRVKVQAAAGTQGGLDPAVVVPDSENMPKDLSDIELLKDPPPGPLDVYRKQASFDWKKMRLFMD-GEEIIRYKNMIWTTLEKDPLF-SQPMVTE-TVEKQRESTFYR----CKRLFEYNFL : 117
PY.11077.9.ACOX3       : MGDRRVKFQASSGTQEGLDPAVVVPGSENMPKDLSDIELLKDPPPGPLDVYRNQASFDWKKMRLFMD-GEEIVRYKNMIWTTLEKDPLF-SQPMVTE-TVEKQRESTFYR----CKRLFEYNFL : 117

                                                                                                                  Fatty acyl CoA oxidase domain
HSA.009223.2.ACOX1     : DPDEIMWFKKLHLVNFVEPVG-----------LNYSMFIPTLLNQGTTAQKEKWLL-----SSKGLQIIGTYAQTEMGHGTHLRGLETTATYDPETQEFILNSPTVTSIKWWPGGLGKTSNHAI : 188
MUS.001258827.1.ACOX1  : DPEEIMWFKKLHMVNFVEPVG-----------LNYSMFIPTLLNQGTTAQQEKWMH-----PSQELQIIGTYAQTEMGHGTHLRGLETTATYDPKTQEFILNSPTVTSIKWWPGGLGKTSNHAI : 188
CF.64869.12.ACOX1      : DDWDKFFYTEAAACHENSPIG-----------LHTAMFLPTLRKQASEDQKKKFLS-----LAENMTIIGTYAQTELGHGTFIRGLETTATYDPKTQEFILHSPTLTSMKYWPGNLGKTVNFCM : 197
CF.11051.3.1.ACOX1     : EEFDKYYYNEAVAPNENNPVG-----------LHFGMFIPTIEKHGTEEQKKKFVQ-----PAKDLKILGTYVQTELGHGTFIRGLETTATFDPKTQEFVMNTPTLTATKYWPGNLGKTVNYCI : 199
CF.17615.31.ACOX1      : DNEELFIIRQALGPNEYYALG-----------VHGFMFIPTIQKLGTEEQKRKWLP-----LAKNLEIIGTYAQTELGHGTFLRGLETTATYDPKTQEFVLNSPTITSMKYWPANLGKTSNYCV : 193
CF.3421.13.ACOX1       : NDKDKLTYKEWVSLKHPYPTTL----------VPHNRLLRKLESYGVQCNAHKWIESFRSSSTQQVTLDGECSRT------FVRGLETTATYEPKTREFVLNSPTLTSMKYWPGHLGKTVNFCI : 197
PY.8373.9.ACOX1        : DEFDKFYYNEATAPNENNPVG-----------LHFGMFIPTIEKHGTEEQKKKFVQ-----PAKDLKIIGTYVQTELGHGTFIRGLETTATFDPKTQEFIVDTPTLSATKYWPGNLGKTVNYCI : 199
PY.9399.9.ACOX1        : DERDKLNYKEAGANHESSPIG-----------VHLSMFLPTLRKQGTEDQRKRYLP-----LAEKLAIIGTYAQTELGHGTFVRGLETTATYDAKTREFVLNSPTLTSMKYWPGHLGKTVNFCI : 197
PY.11121.10.ACOX1      : DEWDQYFYTEAAACHENSPIG-----------LHSSMFLPTLRKQASEDQKKKFLP-----LAENMTIIGTYAQTELGHGTFIRGLETTATYDSKTQEFILHSPSLTSMKYWPGNLGKTVNFCI : 191
PY.5547.59.ACOX1       : DQEECFIIRQAIGPNEYYAIG-----------VHGFMFIPTIQKLGTEEQKRKWLP-----LAENLTIIGTYAQTELGHGTFIRGLETTATYDPKTQEFVLNSPTITSMKYWPANLGKTSNYCV : 193
HSA.003491.1.ACOX2     : EDGRELGYA-YRALSGDVALN-----------IH-RVFVRALRSLGSEEQIAKWDP-----LCKNIQIIATYAQTELGHGTYLQGLETEATYDAATQEFVIHSPTLTATKWWPGDLGRSATHAL : 203
MUS.001155139.1.ACOX2  : EDGPERIYAD-RVLAGYNNLN-----------LH-GIAMNAIRSLGSDEQIAKWGQ-----LGKNFQIITTYAQTELGHGTYLQGLETEATYDATTQEFVIHSPTMTSIKWWPGDLGRTVTHAV : 203
CF.52871.92.ACOX2      : EDDLHVA---LRIIRDQNCLT-----------LHHSMFLPALERLASDEQKARWLP-----LARRYNMVGTYAQTELGHGTNLMELETTATYDKSTDEFVLSTPKLSSIKWWPGSLGKTSNHAI : 198
PY.716361.21.ACOX2     : EDDLLLA---LRLIRDQNCLS-----------LHHSMFLPALERLASDDQKARWLP-----LARRYHMVGTYAQTELGHGTNLMELETTATYDKATDEFVLSTPKLSSIKWWPGSLGKTSNHAI : 198
HSA.O15254.ACOX3       : SVEDMFK-SPLKVPALIQCLGMYDSSLAAKYLLHSLVFGSAVYSSGSE-RHLTYIQ-----KIFRMEIFGCFALTELSHGSNTKAIRTTAHYDPATEEFIIHSPDFEAAKFWVGNMGKTATHAV : 208
MUS.109646.2.ACOX3     : KVEELLK-NPLKILVLINCLGMYDWSLANKCVLHMLVFGTTVFVSGSE-KHFKYLE-----KIYSLEIFGCFALTELSHGSNTKAMRTTAHYDPDTQEFILHSPDFEAAKFWVGNLGKTATHAV : 208
CF.57427.4.ACOX3       : SEEALFD-NPLKHKILTDCLGMYNWSLGAKYQLDVEMTGGTVQATGSA-RHADIVE-----KIKNFEYFGCFALTELSHGSNTKAMRTTATYDLKTEEFILQTPDFEATKIWSGNMGKMATHAI : 234
PY.11077.9.ACOX3       : SEDALFD-NPLKHKILTDCLGMYNWSLGAKYQLDVEMTGGTVQATGSA-RHGDIVE-----KIKSFEYFGCFALTELSHGSNTKAMRTTATYDPKTEEFVLQTPDFEATKIWSGNMGKMATHAI : 234

HSA.009223.2.ACOX1     : VLAQLIT-KGKCYGLHAFIVPIREIGTHKPLPGITVGDIGPKFGYDEIDNGYLKMDNHRIPRENMLMKYAQVKPDGTYVKPLS--NK---LTYGTMVFVRSFLV-GEAARALSKACTIAIRYSA : 305
MUS.001258827.1.ACOX1  : VLAQLIT-RGECYGLHAFVVPIREIGTHKPLPGITVGDIGPKFGYEEMDNGYLKMDNYRIPRENMLMKYAQVKPDGTYVKPLS--NK---LTYGTMVFVRSFLV-GSAAQSLSKACTIAIRYSA : 305
CF.64869.12.ACOX1      : IMAQLYT-KGKCYGVHAFLLPLRDLETHQSLPGIELGDIGPKFGYGTNDNGYLRLNNVRIPRDHMLMRYSKVLEDGTYVKPPN--SR---LSYGSMTLVRAAIV-GDSSRGLAQACTISIRYSA : 314
CF.11051.3.1.ACOX1     : IMAQLYS-QGKCQGPHMFLCPLRDLNTHQSLPGVELGDIGPKFGYAANDNGYLRLNNLRIPHDHMLMRYSKVLEDGTYVKPAN--SK---LSYGSMVMIRASIV-GFSSRALAQACVIAVRYSA : 316
CF.17615.31.ACOX1      : IMAVLNT-QGKNHGLHSFMVPLRDLKTHESLPGVEVGDLGPKFGFNENDNGFLRLHNVRIPRDHMLMRYAKVSEDGTFTVSASEGSR---LTYGTMLFVRAMLV-TGTSDVLAMACTVATRYSA : 312
CF.3421.13.ACOX1       : VMAQLYT-NGKCYGMHTFLLPLRDLRTHRSLPGIELGDIGPKFGYSSNDNGYLILNNVRIPRENMLMRYAQVLEDGTYVKPSN--AK---ISYGTMTLIRVGIV-TNVSRCLAQACVIAVRYSA : 314
PY.8373.9.ACOX1        : LMAQLYS-QGKCQGPHMFLCPLRDLTTHQSLPGVELGDIGPKFGFNANDNGYLRLNNFRIPRINMLMRYSKVLEDGTYVKPAN--SK---LSYGSMVMIRASIV-GAVSRALAQACVISVRYSA : 316
PY.9399.9.ACOX1        : VMAQLYT-NGKCYGMHTFLLPLRDLSTHQSLPGIELGDIGPKFGYGSNDNGYLILNNVRIPRENMLMRYAQVLEDGSYVKPAN--AK---ISYGTMTLIRVGIV-TNVSRCLAQSCVIAVRYSA : 314
PY.11121.10.ACOX1      : IMAQLYT-KGKCHGIHTFLLPLRDLQTHQSLPGIELGDIGPKFGYGTNDNGYLRLNNVRIPRDYMLMRYSKVLEDGTYVKPPN--SR---LSYGSMTLIRAAIV-GDSSRGLAQACTISIRYSA : 308
PY.5547.59.ACOX1       : IMAVLNT-QGKNHGLHSFMVPLRDLKTHKSLPGVEVGDLGPKFGFNENDNGFLRLHHVRIPRDHMLMRYAKVAEDGAFTVSTNEGSR---LTYGTMLFVRAMIV-SGTSTVLGMACTVATRYSA : 312
HSA.003491.1.ACOX2     : VQAQLIC-SGARRGMHAFIVPIRSLQDHTPLPGIIIGDIGPKMDFDQTDNGFLQLNHVRVPRENMLSRFAQVLPDGTYVKLGT--AQ---SNYLPMVVVRVELLSGEILPILQKACVIAMRYSV : 321
MUS.001155139.1.ACOX2  : VLAHLIC-LGARHGMHAFIVPIRSLEDHTPLPGITVGDIGPKMGFENIDNGFLRLNHVRVPRENMLSRFAEVLPDGTYQRLGT--PQ---SNYLGMLVTRVQLLYKGFLPTLQKACTIAVRYAV : 321
CF.52871.92.ACOX2      : VLAQLVVDNKKC-GMHPFMVQLRSMIDHTPMPGIKLGVIGQKMGGNYNDNGFLFLNNVRIPRENMLMKNAQVTRTGKFLTSGP--NK---ANYATMIYVRVMIL-SWSFGAICAAATAAIRYSS : 315
PY.716361.21.ACOX2     : VLAQLVVDSKKC-GMHPFMVQIRSLVDHTPMPGIKLGVIGQKMGSNHNDNGFLFLNNVRIPRENMLMKNAQVTRAGKFLTSGP--NK---ANYATMIYVRVMIL-SWSFGAISAAATAAIRYSS : 315
HSA.O15254.ACOX3       : VFAKLCVPGDQCHGLHPFIVQIRDPKTLLPMPGVMVGDIGKKLGQNGLDNGFAMFHKVRVPRQSLLNRMGDVTPEGTYVSPFKDVRQRFGASLGSLSSGRVSIV-SLAILNLKLAVAIALRFSA : 331
MUS.109646.2.ACOX3     : VFAQLYMPDGQCHGLHSFLVQIRDTKTLLPMTGVMVGDIGKKLGQNGLDNGFAMFNKVRIPRQNLLDRTGNITSEGTYNSPFKDVRQRLGASLGSLSSGRISII-SMSVVNLKLAVSIAIRFSA : 331
CF.57427.4.ACOX3       : TYAQLYTPDGNCHGLHSFIVPIRDPNTLLAYPGVIVGDMGEKLGQNGLDNGFMAFNNYHIPRETLLNRTGDIKPDGTYVTPYKDPSKRFGASLGALSGGRVGIT-GMGTCNLKLCMPIAIRYSA : 357
PY.11077.9.ACOX3       : VYAQLYTPDGSCHGLHSFIVPFRDPNTLLAYPGVIVGDMGEKIGQNGLDNGFMAFNGYHIPRETLLNRTGDVKADGTYVTPYKDPSKRFGASLGALSGGRVGIT-GMGTCNLKLCMPIAVRYSA : 357
```

```
                                                                                                        FAD-binding motif          *
HSA.009223.2.ACOX1       : VRHQSEIKPGEPEPQILDFQTQQYKLFPLLATAYAFQFVGAYMKETYHRINEGIGQGDLSELP-----ELHALTAGLKAFTSWTANTGIEACRMACGGHGYSHCSGLPNIYVNFTPSCTFEGEN : 424
MUS.001258827.1.ACOX1    : VRRQSEIKRSEPEPQILDFQTQQYKLFPLLATAYAFHFLGRYIKETYMRINESIGQGDLSELP-----ELHALTAGLKAFTTWTANAGIEECRMACGGHGYSHSSGIPNIYVTFTPACTFEGEN : 424
CF.64869.12.ACOX1        : VRRQTEVSPGS-----------IYMMSPLYSI------------------------------------QLHALSAGLKAFTSWECSSGIEVCRMACGGHGYSHASGLPKLYLDLTPGCTYEGEN : 391
CF.11051.3.1.ACOX1       : VRRQTEVSPGGPEAQILDYQTQQYKLFPLIATSYAYHFAGQAVQSNFLKISKEIEAGNLQQMP-----VLHALSAGLKAFSSWGASAGIEVCRMSCGGHGYSMASGLPKIYTNITPACTYEGEN : 435
CF.17615.31.ACOX1        : VRRQTEVSPGGPEAQIMDYQTQQYKLFPLIATTFAFRFTGQSIFNYYTTIDANIEKGQLDDLP-----QLHALASGLKAFCTSVAADGVETCRLACGGHGYSHASGLPWMYTKATVECTYEGEN : 431
CF.3421.13.ACOX1         : VRRQTEVSPGGPEAQILDYQTQQHKLFPLISTAFAFYLTGIEVYKIYNMVNNNVEQGNLDQIG-----ELHGLSAGLKAFSSSSCCVGIEVCRLACGGHGYSHASGLPKIYVNAIPNCTYEGEN : 433
PY.8373.9.ACOX1          : VRRQTEVSPGGPEAQILDYQTQQYKLFPLVATAYAYHFAGQAVQSNFLNISKEIEAGNLQQMP-----VLHALSAGLKAFSSWGASAGIEVCRMSCGGHGYSLASGLPKIYTNITPACTYEGEN : 435
PY.9399.9.ACOX1          : VRRQTEVSPGGPEAQILDYQTQQHKLFPLISTAFAIYLTGIEVYRIYNGVNNNVEQGNLDQIG-----ELHGLSAGLKAFTSSSCCLGIEVCRLACGGHGYSQASGLPKIYVNAIPNCTYEGEN : 433
PY.11121.10.ACOX1        : VRRQTEVYPGGPEAQIMDYQTQQYKLFPLLATAYAFHFTAQMVYGIYLQVTSQIEEGNLDQIG-----ELHALSAGLKAFTSWECSSGIEVCRMACGGHGYSHASGLPKIYVDLTPGCTYEGEN : 427
PY.5547.59.ACOX1         : VRRQTEVSPGGPEAQIMDYQTQQYKLFPLIATAYAFRFTGHSIFNYYTTINANIEKGNLEDLP-----QLHALAAGLKAFCTSVAADGVEICRLACGGHGYSHASGLPWMYTKATVECTYEGEN : 431
HSA.003491.1.ACOX2       : IRRQSRLRPSDPEAKVLDYQTQQQKLFPQLAISYAFHFLAVSLLEFFQHSYTAILNQDFSFLP-----ELHALSTGMKAMMSEFCTQGAEMCRRACGGHGYSKLSGLPSLVTKLSASCTYEGEN : 440
MUS.001155139.1.ACOX2    : IRHQSRLRPSDPEAKILEYQTQQQKLLPQLAVSYALHFMTTSLLQFFHSSYSDILKRDFSLLP-----ELHALSTGMKAMSSDFCAQGTEICRRACGGHGYSKLSGLPTLVTQAIASCTYEGEN : 440
CF.52871.92.ACOX2        : VRRQCSLASGQEESQVLDYQTQQYKLFPPLASGYGFFFIHKVLSAKYSAMLVDLQSGNSKALP-----EMHAWSSGMKALITDITTFNMEICRRSCGGHGYLRASGLSDMVCGAVPLATVEGEN : 434
PY.716361.21.ACOX2       : VRRQSSLAPGQEESQVLDYQTQQYKLFPTLASGYGFFFIHKVLSAKYKAMLVDMQSGNSKALP-----EMHAWSSGMKALITDITTYNMEICRRSCGGHGYLRASGLSDMVCEAFPLATVEGEN : 434
HSA.O15254.ACOX3         : TRRQFG-PTEEEEIPVLEYPMQQWRLLPYLAAVYALDHFSKSLFLDLVELQRGLASGDRSARQAELGREIHALASASKPLASWTTQQGIQECREACGGHGYLAMNRLGVLRDDNDPNCTYEGDN : 454
MUS.109646.2.ACOX3       : TRCQFG-PTDKEEIPVLEYPLQQWRILPYLAAAYALDHFSKTIFMDLIEVQSARLRGDHSDQQAELGREIHALASAGKPLASWTAQRGIQECREACGGHGYLAMNRFGDLRNDNDPNCTYEGDN : 454
CF.57427.4.ACOX3         : VRRQFG-PVGGEEIPVLEYQLQQWRLIPYLAATYALEHFSDSFFMDFVGIRVGLLLGDNSNWQAELGREIHALSCASKPFSAWVARDAIQECREACGGHGFFKVNRLGEIRDDHDPNCTYEGDN : 480
PY.11077.9.ACOX3         : VRRQFG-PAGGEEIPVLEYQLQQWRLIPYLAATYALEHFSDSFFMDFVGIRVGMLLGDKSDWQAELGREIHALSCASKPLSAWIARDAIQESREACGGHGYFKVNRLGEIRDDHDPNCTYEGDN : 480

HSA.009223.2.ACOX1       : TVMMLQTARFLMKSYD-QVHSG------KLVCGMVS---YLNDLPSQRIQPQQVAVWPTMVDINSPESLTEAYKLRAARLVEIAAKNLQKE-VIHRKSKEVAWNLTSVDLVRA-SEAHCHYVVV : 536
MUS.001258827.1.ACOX1    : TVMMLQTARFLMKIYD-QVQSG------KLVGGMVS---YLNDLPSQRIQPQQVAVWPTLVDINSLDSLTEAYKLRAARLVEIAAKNLQAQ-VSHRKSKEVAWNLTSVDLVRA-SEAHCHYVTV : 536
CF.64869.12.ACOX1        : TVMMLQTARYLMKCFS-KVENG------EKLPGFVS---YLST---------DLSVKSCMKSDVSLDCLVKAFEHRATRLVQVAARNIKRLVVEEGQSQQEAWNNTAQQLVWA-SRAHCHTYVV : 495
CF.11051.3.1.ACOX1       : TVMMLQTARYLLKCAL-MVQKG------QRLPDLVA---YLST---------DLSAKSCMTSDVALGCLVKAYQHRAARLTKAATAAVQRY-TSEGKNPTDAFNMASTQLSWA-ARAHCHQYVV : 538
CF.17615.31.ACOX1        : TVMFLQTARFLMKCYN-QLKMK------KSLPSSFA---YITD---------KLSGKSDLDNKLNFNSIIQAYEHRAARLIKAAALNISFW-VKAGVKPWDSTNKSSIHMVWA-AKASCHLFVV : 534
CF.3421.13.ACOX1         : TVLYLQTARYLIKCFT-KVQKG------EKLPGFVS---YMGT---------DLTIKSTIKSDVALESIVLAYEHRAARMVRAAAMNIQQL-VREGFTQQEAWNKSTQQLVWA-TMAHCHAYVV : 536
PY.8373.9.ACOX1          : SVMMLQTARYLFECAM-MVQKG------QRLPDLVS---YLSM---------DLSAKSCMTSDVALGCLVKAYQHRAARLTNAATAVVQRH-INEGQKPTDAFNMASTQLTWA-ARAHCHQYVV : 538
PY.9399.9.ACOX1          : TVLYLQTARYLMKCFT-KVQMG------EKLPGFVS---YLGT---------DLSIKSTIKSDVSLESLLLAYEHRAARMVKAAAMNIQGL-VQEGFTQQEAWNKSTQQLVWA-TMAHCHTYVV : 536
PY.11121.10.ACOX1        : TVMMLQTARYLMKCFA-KVENG------EKLPGFVS---YLNT---------DLSAKSCMKSDVALDCLVQAYEHRAARLVQVAARNIKRLVVEEGQSQQQAWNNTAQQLVWA-SRAHCHTYVV : 531
PY.5547.59.ACOX1         : TVMFLQTARYLVKCYN-QMKMK------KSLPSSFA---YITD---------NLSGKSDLDNKLNFDSIIEAYEHRAARLIKAAALNITFW-MKAGNKVWDSTNKSSIQMVWA-AKASCHLFVV : 534
HSA.003491.1.ACOX2       : TVLYLQVARFLVKSYL-QTQMSPGSTPQRSLSPSVA---YLTA---PD---LARCPAQRAADFLCPELYTTAWAHVAVRLIKDSVQHLQTL-TQSGADQHEAWNQTTVIHLQA-AKVHCYYVTV : 552
MUS.001155139.1.ACOX2    : TVLYLQVARFLMKSYL-QAQVSPGSIPQKPLPQSVM---YLAT---PR---PARCPAQTAADFRCPEVYTTAWAYVSARLIRDATQHTQTL-MRSGVDQYDAWNQTSVIHLQA-AKAHCYFLTV : 552
CF.52871.92.ACOX2        : TVLYLQVARYLLKQIA-KALTG------HQTTGTTE---YLNM--NEA---SYICPLQKPVDCRNTEFLCEVYKKRAYRMVLKVAQALQQD-MEAGKEQAVYMNNNLQPLVRM-ATAHCYYLAM : 541
PY.716361.21.ACOX2       : TVLYLQVARYLLKQIA-KALTG------QQTTGTTE---YLTM--NEA---SYICPLQKPVDCRNPDFLCEVYKKRAYRMVLKVAQALQRD-MESGKEQAVYMNNNLQPLVRM-ATAHCYYLAM : 541
HSA.O15254.ACOX3         : NILLQQTSNYLLGLLAHQVHDG------ACFRSPLKSVDFLDAYPGIL---DQKFEVSSVADCLDSAVALAAYKWLVCYLLRETYQKLNQE-KRSGSSDFEARNKCQVSHGRPLALAFVELTVV : 568
MUS.109646.2.ACOX3       : NVLLQQTSNYLLSLLEPPLQDG------AHFTSPLKTVDFLEAYPGIL---GQKFLGSSKADWMDSAAPLAAYRWLVCYLLQESHRRYCQE-KKSRGSDFEARNNSQVYGCRPLALAFMELTVM : 568
CF.57427.4.ACOX3         : NVILQQTSNYLLGLLESNIKNG------EEINTPLKSVNFLSDLDGLL---KTTFEARTVEDCFNPEVSLRAYKWLVGRLLVDSAERLKEQ-GQAGKDAFSARNDSQAYYCRSLALAYIEHTVL : 594
PY.11077.9.ACOX3         : NVILQQTSNYLLGLLESNIRRG------EAINTPLKSVNFLSDLDGLL---QTTFEARTVEDCFNPEVSLRAYKWLVGRLLVDSAERLKEQ-GQAGKDAFSARNDSQAYYCRSLALAYIEHTVL : 594

HSA.009223.2.ACOX1       : KLFSEKLLK---IQ-DKAIQAVLRSLCLLYSLYGISQNAGDFLQGSIMTEPQI-TQVNQRVKELLTLIRSDAVALVDAFDFQDVTLGSVLGRYDGNVYENLFEWAKNSP--LNKAEVHESY-KH : 652
MUS.001258827.1.ACOX1    : KVFADKLPK---IQ-DRAVQAVLRNLCLLYSLYGISQKGGDFLEGNIITGAQM-SQVNSRILELLTVTRPNAVALVDAFDFKDVTLGSVLGRYDGNVYENLFEWAKKSP--LNKTEVHESYYKH : 653
CF.64869.12.ACOX1        : KTFVSMVTE---SNFDDQVLNVLTSLCKLYAVHGIMENLGEFIQDGYLSTSQV-EDVTGKLMVLMKEIRPDAVALVDAFDYPDAVLGSCLGRYDGNVYQALYDYAKSSP--LNKTDVLDTYEKY : 613
CF.11051.3.1.ACOX1       : KVFVTMVTE---AKIDDRTKAALTTLCKMYAVNGIMENLGEFLQDQFFNADQV-DILTGKMMTLLADVRPDAVALVDAFDFHDKVLDSCLGRYDGNVYYALYEYAKSSP--LNEKDVLDSFHKY : 656
CF.17615.31.ACOX1        : KTFINVVTS---ANLDPKVRSALTTLCRLYAVNGMIENMGQFSQDGFLDETQI-NMLNRRLTDLLEEVRTNAVTFVDAFEYHDNELQSCIGRYDGQVYEALYNYAKSSP--LNDTDVHSSFHKY : 652
CF.3421.13.ACOX1         : KTFTARITR---SDLDDKTKKVMTALCKLYGVHGMVENLGDFIQDGFMNASQV-DAVTRKLMFLLAEIRPDAVALVDAFDYPDKVLDSCLGRYDGDVYKALYEYAKASP--LNDKDVLDSFNTY : 654
PY.8373.9.ACOX1          : KVFVTTVTE---ANIDDRTKAALTTLCKMYAVNGIMENLGEFLQDQFFNTDQV-DILTRKMMTLLADVRPDAVALVDAFDYHDKVLDSCLGRYDGNVYYALYEYAKSSP--LNEKDVLDSFHKY : 656
PY.9399.9.ACOX1          : KTFTARITG---SNLDDQTKKVMTALCKLYGVHGMVENLGDFIQDGFMNASQV-DTVTRKLMYLLAEIRPNAVALVDAFDYPDQVLDSCLGRYDGDVYKALYEYAKASP--LNNKDVLDSFNTY : 654
PY.11121.10.ACOX1        : KAFVAMVTE---SNLDDQVLNVLTSLCKLYAVHGVMEHLGEFIQDGYLSTSQV-EAVTGKLMALMKEIRHNAVALVDAFDYPDALLSSCLGRYDGNVYQALYDYAKSSP--LNQKDVLDSYEKY : 649
PY.5547.59.ACOX1         : KTFINLVTS---ANLDPKVRTALTTLCRLYAVNGMIENMGQFSQDGFLNETQI-NMLNGRLSALLEEVRANAVAFVDAFEYHDNELHSCLGRYDGQVYEALYKYAKSSP--LNDTDVHSSFHKY : 652
HSA.003491.1.ACOX2       : KGFTEALEK---LENEPAIQQVLKRLCDLHAIHGILTNSGDFLHDAFLSGAQV-DMARTAYLDLLRLIRKDAILLTDAFDFTDQCLNSALGCYDGNVYERLFQWAQKSP--TNTQE-NPAYEEY : 669
MUS.001155139.1.ACOX2    : RNFKEAVEK---LDNEPEIQRVLQNLCDLYALNGILTNSGDFLHDGFLSGAQV-DMARTAFLDLLPLIRKDAILLTDAFDFSDHCLNSALGCYDGHVYQRLFEWAQKSP--ANTQE-NPAYKKY : 669
CF.52871.92.ACOX2        : AGYVEGLAS---VQLSPPAKKILNKLCCLFGVHYIVTLSGDFLEAEAIGLEQL-QWLQDLEIQLLAEIRPDAVTLVDALDFHDETLSSAIGCYDGRAYERLYEAALKEP--MNQTEVDPAYEKY : 659
PY.716361.21.ACOX2       : RSYIEGLAS---LQVSPPARKILDKLCCLFGVHYIVTLSGDFLEAEAIGLEQL-QWLQELELQLLAEIRPDAVTLVDALDFHDETLSSAIGCYDGRAYERLYEAALKEP--MNQTEVDPAYDKY : 659
HSA.O15254.ACOX3         : QRFHEHVHQ---PSVPPSLRAVLGRLSALYALWSLSRHAALLYRGGYFSGEQAGEVLESAVLALCSQLKDDAVALVDVIAPPDFVLDSPIGRADGELYKNLWGAVLQESKVLERASWWPEFSVN : 689
MUS.109646.2.ACOX3       : QRFHEHIHS---SGLSPSLRTVLGRLSTLYGLWCLSQHMALLYRGGYISGEQTGRAMEDAILTLCEQLKDDAVALVDVIAPSDFVLNSPIAKADGELYKNLWAAVLQQNGVLERAAWWPEFSAN : 689
CF.57427.4.ACOX3         : DRFHQLETEGRNGDTPPELKPVLNKMCALFGLWRLEKHLSTLYQGGYIKGSNPPKLIQEAILKLCYSLKGEAVSLVDAIAPTDFVLNSPIGLSDGQIYKNLYGAMIQGPNALERPVWWKDFVDN : 718
PY.11077.9.ACOX3         : DRFHQLEMEGRNGDTPPELKPVLNKMCALFGLWRLEKHLSTLYQGGYIKGSNPPKLIQEAILKLCYALKGEAVSLVDAIAPTDFVLNSPIGLSDGQIYKNLYGAMIQGPNALERPAWWKDFVDN : 718
```

peroxisomal targeting signal

```
HSA.009223.2.ACOX1          : LKSLQ------------SKL : 660
MUS.001258827.1.ACOX1       : LKPLQ------------SKL : 661
CF.64869.12.ACOX1           : LKPMRGDTSTYPLRA--SKL : 631
CF.11051.3.1.ACOX1          : IKPMREGQATSSLP---ARL : 673
CF.17615.31.ACOX1           : LAPLKKE----------SKL : 662
CF.3421.13.ACOX1            : LRPLQGNMTTSTF----AKL : 670
PY.8373.9.ACOX1             : IKPLREGQATSSLP---SRL : 673
PY.9399.9.ACOX1             : LRPLQGNTGTSSL----AKL : 670
PY.11121.10.ACOX1           : LKPMRGDTSNSAHPLRASKL : 669
PY.5547.59.ACOX1            : LAPLKKE----------SKL : 662
HSA.003491.1.ACOX2          : IRPLLQSWR--------SKL : 681
MUS.001155139.1.ACOX2       : IRPLMQSWK--------PKL : 681
CF.52871.92.ACOX2           : LKHLIMEGAP-------SKL : 672
PY.716361.21.ACOX2          : LKHLIMEGAQ-------SKL : 672
HSA.O15254.ACOX3            : -KPVIGSLK--------SKL : 700
MUS.109646.2.ACOX3          : -KSVADRLK--------SQL : 700
CF.57427.4.ACOX3            : -KPVVGSK---------SKL : 728
PY.11077.9.ACOX3            : -KPVVGSK---------SKL : 728
```
